# Retention of HIV-Infected Children in the First 12 Months of Anti-Retroviral Therapy and Predictors of Attrition in Resource Limited Settings: A Systematic Review

**DOI:** 10.1371/journal.pone.0156506

**Published:** 2016-06-09

**Authors:** Lisa L. Abuogi, Christiana Smith, Elizabeth J. McFarland

**Affiliations:** 1 Department of Pediatrics, University of Colorado School of Medicine and Children’s Hospital Colorado, Aurora, Colorado, United States of America; 2 Center for Global Health, University of Colorado School of Medicine School of Public Health, Aurora, Colorado, United States of America; 3 Department of Pediatric Infectious Diseases, University of Colorado School of Medicine and Children’s Hospital Colorado, Aurora, Colorado, United States of America; Infectious Disease Service, UNITED STATES

## Abstract

Current UNAIDS goals aimed to end the AIDS epidemic set out to ensure that 90% of all people living with HIV know their status, 90% initiate and continue life-long anti-retroviral therapy (ART), and 90% achieve viral load suppression. In 2014 there were an estimated 2.6 million children under 15 years of age living with HIV, of which only one-third were receiving ART. Little literature exists describing retention of HIV-infected children in the first year on ART. We conducted a systematic search for English language publications reporting on retention of children with median age at ART initiation less than ten years in resource limited settings. The proportion of children retained in care on ART and predictors of attrition were identified. Twelve studies documented retention at one year ranging from 71–95% amongst 31877 African children. Among the 5558 children not retained, 4082 (73%) were reported as lost to follow up (LFU) and 1476 (27%) were confirmed to have died. No studies confirmed the outcomes of children LFU. Predictors of attrition included younger age, shorter duration of time on ART, and severe immunosuppression. In conclusion, significant attrition occurs in children in the first 12 months after ART initiation, the majority attributed to LFU, although true outcomes of children labeled as LFU are unknown. Focused efforts to ensure retention and minimize early mortality are needed as universal ART for children is scaled up.

## Introduction

In 2014, the Joint United Nations Programme on HIV/AIDS (UNAIDS) outlined ambitious targets to end the HIV epidemic by ensuring that 90% of people living with HIV know their diagnosis, 90% of those diagnosed receive sustained anti-retroviral therapy (ART), and 90% on ART achieve viral suppression (the so-called 90-90-90 goals). [1] In the same year, UNAIDS estimated that there were 2.6 million children under 15 years of age living with HIV, the majority of whom are living in resource limited settings (RLS). [2] In 2013, the World Health Organization (WHO) expanded the number of children recommended to initiate ART, including all those under 5 years of age. [2, 3] In response to results from the Strategic Timing of Antiretroviral Treatment (START) trial demonstrating benefit to early initiation of ART, WHO released rapid advice in September 2015 recommending that all people living with HIV be offered ART. [4–6] Despite these recommendations, only one out of three HIV-infected children is currently receiving ART by best estimates. [2]

In order to achieve 90-90-90 targets for children, HIV-infected children must be identified, linked to care, initiated on ART, retained in care, and finally achieve sustained virologic suppression. [7] However, the HIV treatment cascade is not well characterized in children. A systematic review by Mugglin et al. showed that pre-ART retention (from diagnosis through enrollment to care) varies from 1–60%, but did not document retention of children after ART initiation. [8] Variable pre-ART retention rates raise concerns about achieving successful health outcomes for children living with HIV. While post-ART retention in care has been well described in adults, it has only been systematically reviewed in children in a single report by Fox and Rosen. [9–12] The authors reported a 12 month retention rate on ART of 85% for all children and youth <18 years; however, risk factors associated with attrition were not explored, and children, who have different rates of and risk factors for attrition, were not described separately from adolescents.

Universal ART coverage for children should significantly reduce morbidity and mortality for children living with HIV and simplify pediatric ART service provision. [3, 13] However, these gains can only be achieved if children are retained after ART initiation. [14] Therefore, it is critical to understand current retention rates, particularly in RLS where more than 90% of HIV-infected children live, and to identify and address barriers to pediatric retention. This systematic review describes retention in the first 12 months after ART initiation for children in RLS and explores factors associated with attrition.

## Materials and Methods

### Data Sources

The PubMed and Embase bibliographic databases were searched on 3^rd^ September 2014 and repeated on 18^th^ March 2015 for English language publications reporting on retention/loss to follow up of pediatric HIV-infected patients in RLS from 2000 onward, during scale-up of pediatric ART programs. Search terms included “retention,” “lost to follow up,” “outcomes,” “attrition,” “anti-retroviral treatment,” “HIV,” and “children,” and their variations. References of relevant articles were reviewed and included. The detailed search strategy is shown in the Supplemental Information. (S1 Text)

### Study selection

Study inclusion and exclusion criteria are outlined in Table 1. All studies that included HIV-infected children with median age at ART initiation of <10 years and reported on the number or proportions retained or lost to follow up (LFU) for a minimum of 12 months following ART initiation were included. A maximum median age at ART initiation of <10 years was used for consistency with the WHO guidelines’ pediatric age definition. UNAIDS is also focusing greater attention on disaggregating adolescents from children. [15] Adolescents >10 years have been shown to have poorer retention rates than both younger children and adults. [16] Additionally, factors associated with LFU in adolescents such as migration for work, internal stigma and discrimination, or challenges in finding youth-friendly care may not apply to younger children. [17]

**Table 1:** Study inclusion criteria.

Inclusion Criteria	Exclusion Criteria
HIV-infected children with median age at ART initiation < 10 years	Children were prescribed less than three drug ART
Retention or loss to follow up reported as numbers or proportions	Study reports on mixed cohort and does not disaggregate data by age
Minimum follow up time of 12 months or median ≥1 year	Study includes children initiated on ART prior to 2000
Low, low-middle, or upper-middle income country as defined by the World Bank	Qualitative study without quantitative outcomes
At least 50 HIV-infected children included	Clinical trial
	Study reporting outcomes for HIV-infected adults, adolescents, HIV-exposed infants or pregnant women only

Studies reporting on mixed cohorts of adults, adolescents, pregnant women and/or HIV-exposed children that did not report age-specific retention rates for HIV-infected children were excluded. Qualitative studies and data from clinical trials were excluded as non-representative of routine HIV care in RLS. Those countries defined as low income, lower-middle income, or upper middle income by the World Bank were included as RLS. [18] The literature search and data extraction were performed in duplicate using a standardized data abstraction form by two different investigators (LLA and CS). Any discrepancies in eligibility or data were reviewed and agreed upon by consensus.

In order to assess the quality of the non-randomized studies included in this review, an assessment of methodological quality was undertaken for each study using the modified Newcastle-Ottawa scale (NOS) for cohort studies. [19] Each study is judged on eight items, categorized into three groups: the selection of the cohort (4 stars); the comparability of the cohort (2 stars); and the ascertainment of outcome of interest (3 stars). Stars are awarded for each quality item and are meant to serve as a rapid visual assessment of study quality. A maximum of nine stars may be awarded to the highest quality studies. All included studies compared unexposed (retained) versus exposed (not retained) in the same cohort, used standardized records to ascertain the outcome of interest (retention) and ensured death and LFU were not present at the start of study, thus automatically had 3 stars under cohort selection. Studies with less than 10 sites did not receive a fourth star for selection unless the sites were purposively chosen to be representative. Studies that reported an association between age or time on ART were eligible for one star for each association under comparability. Studies that defined LFU, followed children for a minimum of 12 months, and reported on transfers out (TO) received one star for each component, respectively under outcome. (S2 Table)

### Data extraction and analysis

A standardized data extraction sheet was used to extract inclusion criteria; characteristics of the program (setting, location, country); characteristics of the children (age at ART initiation or baseline, gender); eligibility criteria for ART; definition of LFU; number and proportion of children alive and on treatment, known to have died, TO, or LFU after ART initiation; and methods of attempting to contact children with missed appointments (“defaulter tracing”). The proportion of children retained in care 12 months after ART initiation was defined as the number of children alive on ART plus those TO, less those LFU, discontinued ART, or dead, amongst total children initiated on ART. Attrition was defined as children not retained in care due to LFU or death, excluding those known to have TO. Transfers out were defined independently by each study, but generally included only those children who were documented to be receiving ART at another facility. Summary or pooled estimates were not calculated because the studies reported on diverse pediatric populations from heterogeneous settings, reflecting wide-ranging HIV prevalence, type of HIV programs, and country-specific approaches to HIV care and treatment for children. Predictors of mortality and LFU were extracted with corresponding risk measures or proportions when available.

## Results

### Study characteristics

**Fig 1:**
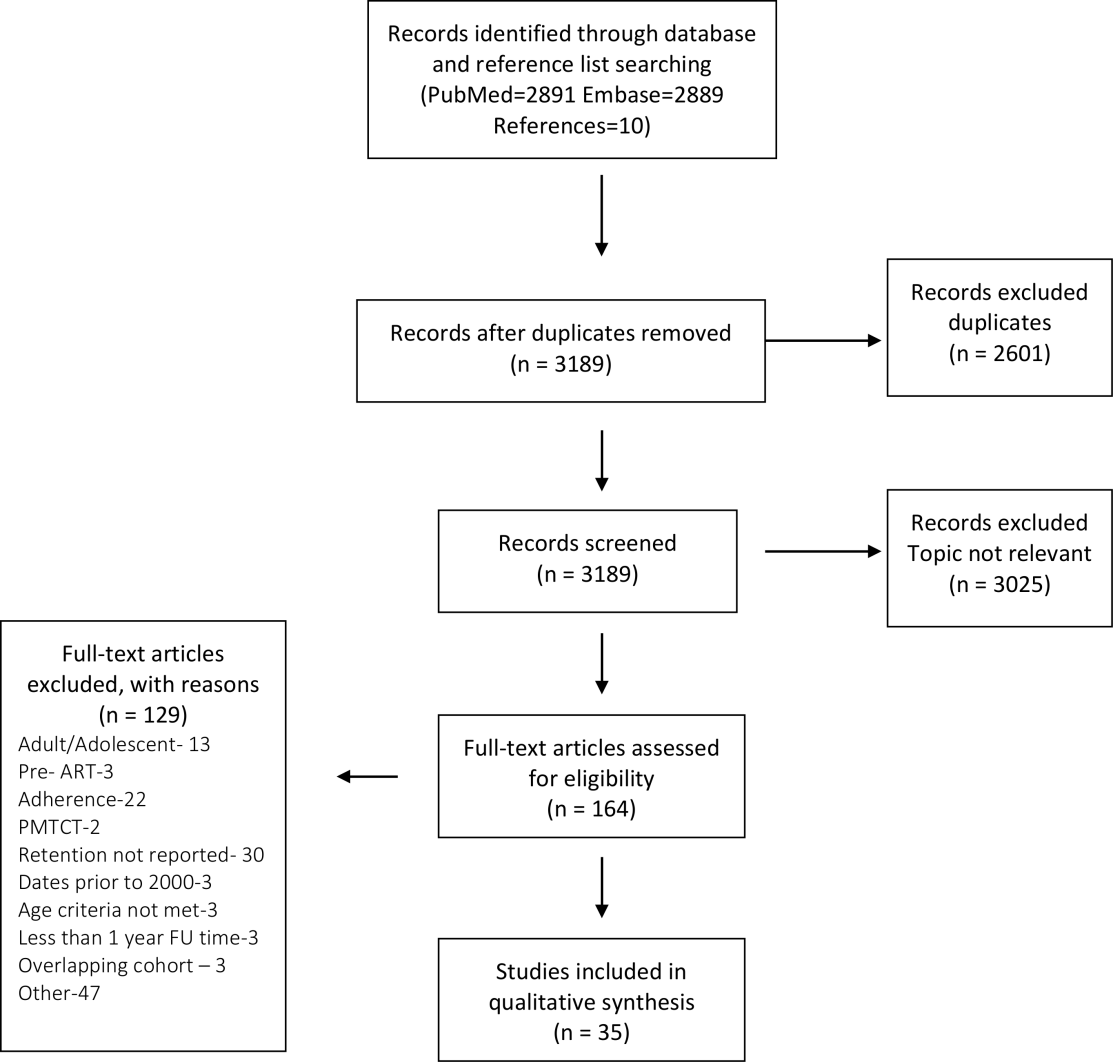
Identification and selection of studies. Fig 1 shows the studies identified and reasons for inclusion or exclusion.

A total of 5,790 articles were identified through electronic bibliographic databases and reference review. (Fig 1) After removing duplicates, 3,189 were screened, identifying 164 full text articles of which 35 published between 2006 and 2014 met inclusion criteria for this systematic review. Overall, 78,424 HIV-infected children on ART from more than 30 countries were represented. The majority of studies 77% (27/35) used a retrospective cohort design. Between one and 380 sites were included: 21 studies included fewer than ten sites, 11 studies 10–100 sites, and 3 studies more than 100 sites. Only one study from Zambia purposively selected 6 sites to represent different health facility levels. [20] Most sites from the included articles were public (government) facilities in urban areas. Detailed site information is available in Table 2.

**Table 2:** Retention of children initiating ART at median age <10 years by overall follow up time.

Total follow up time^a^ (years)	Retention (%) at end of follow up	Location	Author, year	Number of facilities	Setting	Median age (years) at ART initiation	Study period	N	LFU definition (months)
**Africa**									
1	77	South Africa	Eley, 2006[21]	1	urban	1.9	2002–2004	409	NR
1	90	South Africa	Sengayi, 2013[22]	1	urban	4.2	2004–2012	4266	6
1	87	Uganda	Ahoua, 2011[23]	1	rural	5.4	2002–2006	87	2
1	71	West Africa^b^	Ekouevi, 2011[24]	9	urban	5.0	2000–2007	2170	6
1.1	75	Zambia	Bolton-Moore, 2007[25]	18	urban	6.8	2004–2007	2938	1
1.4^c^	92	South Africa	Fatti, 2011[26]	30	both	6.4	2004–2010	3007	3
1.5^c^	90	South Africa	Janssen, 2010[27]	19	rural	6.2	2004–2008	477	3
1.7	86	Africa^d^	KIDS-ART-LINC, 2008[28]	16	urban	4.9	NR-2007	2405	6
1.8	87	Ethiopia	Koye, 2012[29]	1	urban	6.4	2006–2011	549	NR
1.8	88	South Africa	Zanoni, 2011[30]	1	urban	6.0	2003–2009	537	3
2	72	East Africa^e^	Fayorsey, 2013[31]	274	both	3^f^	2008–2010	8475	3
2	72	East Africa^g^	McNairy, 2013[32]	192	both	4.6	2005–2011	17712	6
2	89	Ethiopia	Asfawesen, 2011[33]	1	urban	5.9	2005–2008	188	NR
2	68	Malawi	Weigel, 2010[34]	1	Urban	8	2001–2008	497	3
2	48	Mozambique	Vermund, 2014[35]	17	rural	1.9	2006–2011	753	2
2	94	Rwanda	vanGriensven, 2008[36]	2	both	7.2	2003–2007	315	2
2	91	South Africa	Jaspan, 2008[37]	1	urban	2.2	2002–2006	391	NR
2	83	South Africa	Meyer-Rath, 2013[38]	2	urban	4.0/5.8^h^	2005–2009	288	3
2.2^c^	69	Côte d’Ivoire	Auld,2014[39]	29	both	5.1	2004–2008	2110	3
2.2–3.1^i^	78	Ethiopia	Hagstromer, 2013[40]	6	both	5.0	2007–2012	974	3
2.3 mean	77	Nigeria	Ojikutu, 2014[41]	23	urban	4.1	2002–2011	1516	3
2.3	85	Southern Africa^j^	Kabue, 2012[42]	3	urban	3.1	2004–2009	2306	NR
2.3	91	Uganda	Bakanda, 2011[43]	11	rural	All <10	2004–2009	810	NR
2.6	84	Congo	Edmonds, 2011[44]	2	urban	5.9	2004–2010	619	Other^k^
2.6	93	Rwanda	Tene, 2013[45]	39	both	6.3	2004–2010	2035	6
2.6	76	South Africa	Barth, 2011[46]	1	rural	5.0	2003–2007	101	NR
3	79	Congo	Ditekemena, 2014[47]	3	both	4.7^c^	2007–2012	522	3
3	67	Zambia	Scott, 2013[20]	6^l^	both	4.0	2006–2011	449	3
4.2^c^	94	Uganda	Massavon, 2014[48]	2	urban	6.6	2003–2010	604	3
**Africa and Asia**									
1.5	82	Africa/Asia^m^	Leroy, 2013[49]	54	both	5	2000–2009	13611	6
**Asia**									
1.7	81	Thailand	McConnell, 2010[50]	380	NR	7.3	2000–2007	3409	3
2	93	Cambodia	Isaakidis, 2010[51]	2	urban	6	2003–2007	670	3
2.0	89	China	Zhao, 2013[52]	28^n^	NR	6.2	2005–2010	1818	3
2.9^c^	81	India	Alvarez-Uria, 2014[53]	3	both	5.7	2007–2013	285	6
**Caribbean**									
1.7	81	Haiti	George, 2007[54]	1	urban	6.3	2003–2006	236	NR

ART, antiretroviral therapy; LFU, lost to follow up; NR, not reported

^a^ Follow up time reported as total years, or median, if child-years reported only, mean follow up was calculated

^b^ Benin, Côte d’Ivoire, Gambia, Ghana, Mali and Senegal

^c^ mean

^d^ Benin, Burundi, Côte d’Ivoire, Ghana, Kenya, Rwanda, Uganda, South Africa, Zambia, Zimbabwe

^e^ Kenya, Lesotho, Mozambique, Rwanda, and Tanzania

^f^ median age at subset of 95 sites from author communication

^g^ Kenya, Lesotho, Mozambique, Rwanda, Tanzania

^h^ Two clinic cohorts reported, aggregate age not given

^i^ Two cohorts from hospitals or health clinics reported, aggregate follow up time not given

^j^ Lesotho, Malawi, Swaziland

^k^ withdrew from care or not located by 3 tracking attempts after missed visit

^l^ 6 purposively selected representative sites

^m^ Benin, Burkina Faso, Cameroon, Republic of the Congo, Cote d’Ivoire, Democratic Republic of the Congo, Guinea, Kenya, Liberia, Malawi, Mozambique, Nigeria, Uganda, Zambia, and Zimbabwe. Myanmar (Burma), Cambodia, China, India, and Laos

^n^ Number of provinces, sites not reported

The majority of studies reported ART initiation criteria based on national guidelines, which in turn were based on relevant WHO guidelines at the time of the study. Studies reported from Thailand specified Thai national guidelines using United States Centers for Disease Control clinical staging and Thai-specific CD4 cut-offs. [50] Definition of LFU varied considerably amongst the 27 studies that provided definitions: four defined LFU as 1–2 months since last contact, fifteen as >3 months from last contact, seven as >6 months, and one study defined LFU as no contact after 3 tracing attempts. A minority of studies described efforts to ensure retention or to trace children LFU (12/35, 34%). Of those studies that described tracing procedures, all reported either phone calls or home visits or a combination of the two. Study quality as assessed by NOS ranged from 4–9 stars out of a maximum of 9 possible stars. Overall, the majority (94%) of studies had >5 stars, indicating generally high quality. (S1 Table)

Follow-up time on ART varied from 1 to 4.2 years. Reported retention rates at end of follow up ranged from 48–94%. Table 2 shows retention rates by total follow up time. Retention varied within years of follow up without obvious trend. Sixty-three percent (22/35) of studies showed retention of 80% or greater at the end of study-specific follow-up while more than a third (37%) reported less than 80% retention. Only 26% (nine studies) reported at least 90% retention. A study conducted in Mozambique reported the lowest retention rate (48%) and two studies from Rwanda and Uganda reported the highest retention rates (93 and 94%, respectively). [35, 45, 48]

### Retention in care at 12 months

Among the 35 articles included, 12 (34%) provided 12-month post-ART retention estimates or sufficient information to derive this. (Table 3) These studies included 31877 children, all from African countries. Nearly half of the children were female and median age at initiation of ART was between 1.9 and 7.2 years. Ten studies reported CD4 percentage at time of ART initiation ranging between 11–15%.

**Table 3:** Retention at 12 months on ART amongst HIV-infected children.

12 month retention on ART (%)	Author, year	Location	N	Sex	Age^a^	CD4%^a^	WHO Stage III/IV^a^	LFU	Dead	TO
				(% female)	(years) (median)	(median)	(%)	N (%)	N (%)	N (%)
71	Ekouevi, 2011	West Africa^b^	2170	45	5	13	NR	461 (21)	169 (8)	NR
73	Scott, 2013	Zambia	1334	NR	4	14	NR	290 (22)	67 (5)	NR
78	Auld, 2014	Côte d’Ivoire	2110	46	5.1	11	82	NR	NR	NR
79	McNairy, 2013	East Africa^c^	17712	51	4.6	15	48	2834 (16)	886 (5)	NR
80	Ahoua, 2011	Uganda	87	45	5.4	11	76	8 (9)	3 (3)	6 (7)
80	Eley, 2006	South Africa	409	46	1.9	12	99	19 (5)	63 (15)	46 (11)
85	Ditkemena, 2014	Congo	522	49	4.7^d^	NR	80	NR	NR	NR
85	Meyer-Rath, 2013	South Africa	288	NR	4.0/5.8^e^	12/14	NR	34 (12)	9 (3)	NR
88	Jaspan, 2008	South Africa	259	47	2.2	13	NR	4 (2)	27 (10)	NR
90	Kids-ART-Linc, 2008	Africa	2405	48	4.9	NR	NR	109 (5)	139 (6)	102 (4)
90	Sengayi, 2013	South Africa	4266	49	4.2	15	74	323 (8)	113 (3)	202 (5)
95	vanGriensven, 2008	Rwanda	315	50	7.2	14	40	NR	NR	NR
**71–95%**		**TOTAL**	**31877**					**4082**	**1476**	**356**

LFU, lost to follow up; TO, transfer out; NR, not reported

^a^ At ART initiation

^b^ Benin, Côte d’Ivoire, Gambia, Ghana, Mali and Senegal

^**c**^ Kenya, Lesotho, Mozambique, Rwanda, Tanzania

^d^ mean

^e^ Two cohorts reported, no aggregate age given

Retention on ART at 12 months ranged between 71–95%. Retention was highest in the pediatric cohort from Rwanda (95%) and lowest in a West African cohort (71%). [24, 36] Nine studies including 28930 children specified the number or proportion of children either LFU, dead or TO. Among these, a total of 5558 (19%) children were not retained: 4082 (73%) were reported LFU and 1476 (27%) died. In addition, 4 studies reported that 356 children TO. No study systematically confirmed the final outcome of children LFU, which could potentially include death or transfer of care to another facility. (Table 3)

### Predictors of mortality and LFU at 12 months

#### Young age

All studies that assessed age as a risk factor found that infants and young children were at higher risk of mortality and/or LFU after ART initiation, compared with older children. This finding was consistent across all geographical regions. Infants <1 year were at particularly high risk, with 2 to 12 times higher mortality compared with older children. [21, 22, 32, 40, 42, 49, 50] Several studies reported a gradually decreasing risk of mortality for children as their age at ART initiation increased. [22, 25, 42] Table 4 summarizes predictors of mortality across studies. LFU also occurred at higher rates in younger children, with infants <1 year old having an aHR of LFU ranging from 1.6–3.3. [32, 40, 49]

**Table 4:** Reported predictors of mortality in children on ART in resource limited settings.

**Age at ART initiation**				
**Author, year**	**Country**	**aHR (95% CI)**	**Age at risk (years)**	**Reference age (years)**
Auld, 2014	Cote d'Ivoire	1.7 (1.2–2.4)	<2	5–14
Ditekemena, 2014	Congo	2.8 (1.2–6.5)	≤2	>2
Eley, 2006	South Africa	2.5 (1.5–4.1)	<1	NR
George, 2007	Haiti	2.3 (1.3–3.8)	<1.5	≥13
Hagstromer, 2013	Ethiopia	12 (3.5–41.3)	≤1	>5
Janssen, 2010	South Africa	3.2 (1.2–9.1)	<1.5	≥5
Kabue, 2012	Southern Africa	8.1(4.5–14.6)	<0.5	≥3
		3.4 (2.0–6.0)	0.5 to <1	≥3
		1.9 (1.2–3.2)	1 to <3	≥3
Leroy, 2013	Africa and Asia	2.8 (2.2–3.5)	<1	10–15
		1.5 (1.2–2.0)	1–2	10–15
McConnell, 2010	Thailand	2.4 (1.2–4.4)	<1	>6
McNairy, 2013	East Africa	3.4 (2.6–4.6)	<1	NR
		2.2 (1.7–2.7)	1 to <2	NR
Sengayi, 2013	South Africa	2.0 (1.3–3.3)	<1	3 to <5
Tene, 2013	Rwanda	2.1 (1.3–3.6)	<1.5	≥5
Zanoni, 2011	South Africa	2.7 (1.4–5.2)	<3	3–18
**Immunosuppression**				
**Author, year**	**Country**	**aHR (95% CI)**	**Risk factor**	**Reference**
Auld, 2014	Cote d'Ivoire	2.5 (1.4–4.6)	CD4 <10%	NR
		2.1 (1.1–3.8)	WHO stage III	NR
		2.7 (1.4–5.2)	WHO stage IV	NR
Bolton-Moore, 2007	Zambia	1.8 (1.1–3.1)	CD4 10 to <20%	CD4 ≥20%
		2.1 (1.3–3.6)	CD4 <10%	CD4 ≥20%
Ekouevi, 2011	West Africa	2.1 (1.1–4.1)	CD4 <15%	CD4 ≥15%
		2.5 (1.6–3.9)	WHO stage IV	NR
Eley, 2006	South Africa	1.1 (1.0–1.1)	Per 100 CD4 cell decrease	N/A
		5.3 (2.3–12.3)	WHO stage IV	NR
		1.8 (1.0–3.3)	Viral load >1 million	NR
George, 2007	Haiti	1.8 (1.4–2.8)	CD4 ≤5%	CD4 >5%
Kabue, 2012	Africa (3 countries)	4.4 (2.2–8.7)	WHO stage IV	WHO stage I
KIDS-ART-LINC, 2008	Africa	2.6 (1.3–5.1)	WHO defined severe immunodeficiency	No severe immunodeficiency
Koye, 2012	Ethiopia	2.2 (1.1–4.7)	WHO defined severe immunodeficiency	No severe immunodeficiency
Leroy, 2013	Africa and Asia	3.0 (2.3–3.9)	CD4 <10%	CD4 ≥20%
		2.1 (1.8–2.5)	WHO stage IV or AIDS	Earlier WHO stages
McConnell, 2010	Thailand	2.4 (1.8–3.2)	CD4 <5%	CD4 ≥5%
		1.8 (1.4–2.4)	Clinical stage C	Clinical stage A or N
McNairy, 2013	East Africa	2.8 (1.9–4.0)	CD4 count <100 cells/μL	CD4 count >350 cells/μL
		1.6 (1.3–1.9)	WHO stage IV	WHO stage III
Ojikutu, 2014	Nigeria	7.2 (1.7–30.2)	WHO defined severe immunodeficiency	No immunodeficiency
Tene, 2013	Rwanda	3.6 (1.3–9.6)	WHO stage II	WHO stage I
		2.6 (1.0–6.8)	WHO stage III	WHO stage I
		9.8 (3.5–27.4)	WHO stage IV	WHO stage I
		2.3 (1.7–3.3)	WHO defined severe immunodeficiency	No immunodeficiency
Zanoni, 2011	South Africa	2.4 (1.2–4.7)	CD4 <10%	CD4 >10%
Zhao, 2013	China	2.4 (1.1–5.2)	WHO stage III or IV	WHO stage I or II
**Anemia**				
**Author, year**	**Country**	**aHR (95% CI)**	**Risk factor**	**Reference**
Auld, 2014	Cote d'Ivoire	1.4 (1.0–2.0)	Hgb <8 gm/dL	Hgb≥8 gm/dL
Bolton-Moore, 2007	Zambia	1.6 (1.0–2.4)	Hgb <8 gm/dL	Hgb≥8 gm/dL
Janssen, 2010	South Africa	4.5 (1.6–12.3)	Hgb <8 gm/dL	Hgb≥8 gm/dL
KIDS-ART-LINC, 2008	Africa	2.6 (1.4–4.7)	Severe anemia for age	NR
Koye, 2012	Ethiopia	2.4 (1.3–4.7)	Hgb <10 gm/dL	Hgb≥10 gm/dL
Zhao, 2013	China	2.2 (1.2–3.9)	Hgb <9 gm/dL	Hgb ≥9 gm/dL
**Malnutrition**				
**Author, year**	**Country**	**aHR (95% CI)**	**Risk factor**	**Reference**
Auld, 2014	Cote d'Ivoire	2.4 (1.6–3.4)	WAZ ≤ -2	WAZ > -2
Bolton-Moore, 2007	Zambia	2.5 (1.3–5.0)	WAZ -2 to -3	WAZ >-1
		3.8 (2.0–7.2)	WAZ <-3	WAZ >-1
George, 2007	Haiti	2.1 (1.4–3.1)	WAZ <-3	WAZ >-3
McConnell, 2010	Thailand	2.4 (1.7–3.4)	WAZ -3 to -2	WAZ ≥ -1
		5.6 (3.9–8.0)	WAZ <-3	WAZ ≥ -1
McNairy, 2013	East Africa	2.4 (2.0–2.8)	WAZ ≤ -2	WAZ > -2
Ojikutu, 2014	Nigeria	1.1 (1.0–1.1)	Per 1 point decrease in WAZ	N/A
Tene, 2013	Rwanda	3.2 (1.3–8.1)	WAZ < -3	WAZ > -2
Zanoni, 2011	South Africa	1.7 (1.4–2.0)	Per 1 point decrease in WAZ	N/A
Zhao, 2013	China	9.1 (2.5–33.2)	WAZ < -2	WAZ ≥ -1

aHR, adjusted hazard ratio; Hgb, hemoglobin; N/A, not applicable; NR, not reported; WAZ, weight-for-age z-score; WHO, World Health Organization

#### Health indices of children at ART initiation

Children who started ART at older ages typically had more severe immunosuppression and other indicators of poor health, such as anemia and malnutrition. Each of these factors was found to independently predict mortality in multiple studies (Table 4). Worse clinical disease stage, lower CD4 percentage, and higher viral load were all found to be predictive of mortality. In addition, some studies reported that LFU was associated with severe immunosuppression [27, 40, 47, 49, 50], anemia [39], and malnutrition [22, 39, 40]. This could indicate that many children who are LFU actually die.

#### Duration of time on ART

Several studies identified higher mortality and LFU in the first year on ART, especially in the first six months. Studies reported that 57–64% of deaths occurred within the first 3 months after starting ART [29, 30, 39], 62–83% within the first 6 months [29, 30, 33], and 78–90% within the first 12 months [29, 33, 42]. For children in the Congo, mortality rates of 16.4 per 100 person-years (p-y) compared with 1.8 per 100 p-y during and after the first 3 months of ART, respectively, were reported. [44] In East Africa, high rates of both mortality and LFU were reported in the first 6 months after ART initiation (9.1 and 26.3 per 100 p-y, respectively), which declined to 6.3 and 18.4 per 100 p-y, respectively, at 12 months. [32]

#### Year of ART Initiation

Children from West Africa who initiated ART prior to 2005 had lower mortality and LFU compared to those initiated during 2005 (aHR_mortality_, 1.7, 95%CI 1.0–2.9; aHR_LFU_ 1.7, 95% CI 1.3–2.4) and during or after 2006 (aHR_mortality_ 2.8 95%CI 1.7–4.7; aHR_LFU_ 3.0, 95%CI 2.2–4.0) (p<0.01). [24] Several cohorts of African and Asian children similarly showed increased rates of mortality and LFU in those who initiated ART in later years compared with earlier years. [22, 39, 49]

#### Facility-level predictors

The impact of urban location on mortality and LFU differed between studies. In the Democratic Republic of Congo, mortality and LFU were higher in urban versus non-urban clinics (aHR_mortality_ 7.5, 95%CI 2.8–19.8; aHR_LFU_ 3.4, 95%CI 2.0–5.9). [47] However, a large cohort combining African and Asian countries found higher LFU in non-urban clinics compared to urban sites (aHR 1.7, 95%CI 1.4–1.9). [49] The impact of facility size and level were likewise mixed. In Cote d’Ivoire, smaller facilities had higher mortality and LFU, but in a combined African and Asian cohort, programs with large cohorts (500–800 children) had higher mortality and LFU compared to smaller cohorts (<250 children). [39, 49]

#### Regional Variation

Studies including more than one country revealed considerable variation in mortality and LFU by country and region. Leroy et al. found that West African programs had a 30% higher risk of mortality compared to East African, Southern African, or Asian programs, and both West African and East African programs had more than 3 times higher risk of LFU compared to Asian cohorts (aHR_East Africa_ 3.5, 95%CI 2.6–4.7; aHR_West Africa_ 3.1, 95%CI 2.2–4.3). [49] Compared with children in Rwanda, children in Mozambique had an almost 17-fold higher rate of LFU (aHR 16.8, 95% CI 8.9–32.0) and children in Tanzania had over 2 times higher mortality (aHR 2.6, 95% CI 1.8–3.6). (32) The authors note, however, that Rwanda reported the smallest proportions of young children and severe immunosuppression, while Mozambique reported the highest. Rwanda also had the lowest mortality and LFU rates amongst five African nations including Kenya, Mozambique, Lesotho, and Uganda, but it again reported the lowest HIV prevalence. [31]

## Discussion

This systematic review including more than 30,000 HIV-infected children under 10 years of age shows that between 5–29% of children initiating ART are dead or LFU by 12 months. The majority of attrition appears to be due to LFU (nearly 75%), but a substantial proportion of children die in the first year of ART and an uncertain proportion transfer care elsewhere. Achieving optimal health outcomes and averting deaths for children living with HIV requires life-long retention on ART. Mortality and LFU early after ART initiation threaten the significant potential benefit of ART for children.

The proportions of children who died or TO are likely higher than reported, given that some children recorded as LFU are misclassified deaths or transfers of care. In a Ugandan study of adults, an investigation of a sample of those LFU determined that 29% had died but 60% were in care elsewhere. [55] By contrast, among a sample of 44 HIV- infected children LFU from a large HIV care program in Western Kenya, 16% had died, and over 50% had not returned to care due to disclosure/discrimination fears, travel, cost of transport, or because they were seeking faith healing or traditional therapies. [56] Only 11% had transferred care to another facility. The results of this study are strikingly different from those found in studies ascertaining outcomes for adults LFU. It appears that a large proportion of adults return to care at other facilities; in contrast, a large proportion of children do not return to care. Additional studies are needed to confirm this outcome and further define the barriers preventing children from remaining in care.

Variability in definitions of LFU, poor documentation of TO, and non-standard assessment periods for retention limit the ability to compare retention of children on ART across programs and countries. The duration of time out of care defined as LFU ranged from 1 to 6 months in the articles included in this review. Adoption of a proposed standardized definition of LFU (no healthcare system access for 90 days after last scheduled appointment) developed by a WHO working group will facilitate monitoring over time and comparison between countries and programs. [57] Very few of the studies reported the proportion of children who have TO. A standard approach to documenting patients TO remains a challenge in RLS. Many patients present to a new clinic and report themselves as new patients rather than receive letters of transfer. [57] Ideally, standardized indicators on pediatric retention as recommended by The Inter-Agency Task Team for Prevention and Treatment of HIV Infection in Pregnant Women, Mother and Children should be used for monitoring the proportion of children alive and on ART at 3, 6, 12, 24, and 36 months, disaggregated by age. [14]

Understanding causes of mortality and LFU is key to improving retention of children on ART. Individual predictors of attrition identified in this review include young age (especially <12 months), severe immunosuppression, anemia or malnutrition at ART initiation, and shorter duration of time on ART. High attrition rates in infants are likely driven by high mortality resulting from rapid disease progression in this age group. [58] As demonstrated by the CHER trial, 50% of infants will die by 2 years of age without ART. [13] High mortality rates in the first six months after starting ART are commonly reported in both adults and children; this likely correlates with high levels of immunosuppression at the time of ART initiation. [32, 59, 60] Therefore, older children with disease progression are also at high risk of mortality by the time they initiate ART. Universal ART started early in life for all children living with HIV should reduce severe immunosuppression at ART initiation, thereby reducing mortality. However, early ART will require early diagnosis and enrollment to care. [61]

None of the included studies reported on potentially important psychosocial factors related to attrition such as orphan status, socio-economic status, or costs associated with care. Some predictors of LFU among children depend on the child’s caregivers who might be affected by poor health, low education levels, and inadequate living conditions. Children whose biological mother is their caregiver are at higher risk of LFU, likely due to high mortality rates in the mothers. [22] Thus, ensuring linkage of adult caregivers to care is necessary to achieve high retention rates in HIV-infected children. Studies evaluating psychosocial factors that contribute to mortality and LFU are urgently needed to define potential interventions.

Surprisingly, several studies reported higher rates of mortality and LFU in children who were treated in later years compared with earlier years. Possibly the success of PMTCT programs in reducing new infections in children combined with the expanded availability of ART in more recent years has resulted in a changing demographic of HIV positive children from families that are either more vulnerable or less motivated for treatment, but this finding deserves further exploration.

Country, regional, and facility variation are expected; however, further investigation into factors affecting this geographic variation including HIV prevalence, funding approach, and health system organization would be informative. Rwanda in particular reported consistently lower rates of mortality and LFU, perhaps due to lower HIV prevalence and a highly functional health care infrastructure. Research is needed to evaluate models of care that maximize retention, such as integration of HIV services within existing child health clinics, as called for by the *Melbourne Statement on Young Children Born into HIV-affected Families*. [62]

Retention strategies reported by the studies in this review focus on home visits and phone calls *after* the child had defaulted from care. Proactive use of community health workers has been shown to improve outcomes including retention within the context of prevention of perinatal transmission services but has not been evaluated specifically for children living with HIV. [63] Current retention approaches in adults are focusing on use of mobile technology and text reminders. [64–66] Few studies have reported on the use of text messaging to improve retention of children; however, one study demonstrated improved retention of mother and baby pairs using text messaging. [67] For children whose caregivers are not also in HIV care, it is uncertain if reminder text messaging will improve retention.

Strengths of this review include the large number of children and diversity of countries represented, the systematic approach, and well defined study question. However, several limitations are notable, including limitation to English-language publications, not including conference abstracts which may have increased the number and variety of cohorts described, and the relatively small number of studies that met the inclusion criteria. As noted above, non-standard reporting of retention at different time points, inconsistent definitions of LFU, and failure to document children TO may limit the accuracy and comparability of these results. Given regional and country variations in retention, generalizability to all children living in RLS may not be appropriate. Additionally, most of the studies reported relied on some form of electronic medical record. Since many settings providing routine care for children with HIV do not have electronic records, this may reduce generalizability. The role of publication bias cannot be ruled out. It is possible that studies with higher retention rates were more likely to be published, given the competitive nature of funding for HIV care programs, and an interest in showing the success of pediatric ART care in general. [35]

## Conclusion

While overall retention on ART for children living with HIV at 12 months is high, retention in most RLS will not achieve 90-90-90 targets. Rapid scale up of immediate ART initiation, innovative approaches to improve pediatric retention in care, and routine surveillance of retention using a standardized approach are needed.

## Supporting Information

S1 Table Study quality as assessed by modified Newcastle-Ottowa scale. (DOCX)

S2 Table PRISM CHECKLIST (2009). (DOCX)

S1 Text Detailed Publication Search Strategy. (DOCX)
