# Supplementary material for: Retention of HIV-Infected Children in the First 12 Months of Anti-Retroviral Therapy and Predictors of Attrition in Resource Limited Settings: A Systematic Review
Source: PLoS One. 2016 Jun 9;11(6):e0156506. doi: 10.1371/journal.pone.0156506 (PMC4900559; doi:10.1371/journal.pone.0156506)
Supplement: S1 Table — (DOCX) [file pone.0156506.s001.docx]

**S1 Table. Study quality as assessed by modified Newcastle-Ottowa scale**

| Study  (possible stars) | Selection^a^  (4) | Comparability^b^ (2) | Outcome^c^  (3) | Total stars (9) |
| --- | --- | --- | --- | --- |
| Ahoua | ★★★ |  | ★★★ | 6 |
| Alvarez-Uria | ★★★ |  | ★★★ | 6 |
| Asfawesen | ★★★ | ★★ | ★★ | 7 |
| Auld | ★★★★ | ★★ | ★★ | 8 |
| Bakanda | ★★★★ | ★★ | ★ | 7 |
| Barth | ★★★ |  | ★★ | 5 |
| Bolton-Moore | ★★★★ | ★ | ★★★ | 8 |
| Ditekemena | ★★★ | ★ | ★★ | 6 |
| Edmonds | ★★★ |  | ★★ | 5 |
| Ekouevi | ★★★ | ★ | ★★ | 6 |
| Eley | ★★★ | ★ | ★★ | 6 |
| Fatti | ★★★★ | ★★ | ★★★ | 9 |
| Fayorsey | ★★★★ |  | ★★ | 6 |
| George | ★★★ |  | ★ | 4 |
| Hagstromer | ★★★★ | ★★ | ★★★ | 9 |
| Isaakidis | ★★★ |  | ★★★ | 6 |
| Janssen | ★★★★ | ★★ | ★★★ | 9 |
| Jaspan | ★★★ |  | ★ | 4 |
| Kabue | ★★★ | ★ | ★ | 5 |
| KIDS-ART-LINC | ★★★★ | ★ | ★★★ | 8 |
| Koye | ★★★ | ★★ | ★★ | 7 |
| Leroy | ★★★★ | ★ | ★★★ | 8 |
| Massavon | ★★★ | ★ | ★★ | 6 |
| McConnell | ★★★★ | ★ | ★★ | 7 |
| McNairy | ★★★★ | ★★ | ★★ | 8 |
| Meyer-Rath | ★★★ |  | ★★ | 5 |
| Ojikutu | ★★★★ | ★★ | ★★ | 8 |
| Sengayi | ★★★ | ★★ | ★★★ | 8 |
| Scott | ★★★★ |  | ★★★ | 7 |
| Tene | ★★★★ | ★ | ★ | 6 |
| vanGriensven | ★★★ |  | ★★★ | 6 |
| Vermund | ★★★★ | ★★ | ★★ | 8 |
| Weigel | ★★★ |  | ★★★ | 7 |
| Zanoni | ★★★ | ★★ | ★★★ | 8 |
| Zhao | ★★★★ | ★★ | ★★★ | 9 |

^a^ Compared unexposed (retained) versus exposed (not retained) in the same cohort (1 star), used standardized records to ascertain the outcome of interest (1 star) and ensured death and LFU not present at the start of study (1 star). Representative sites or > 10 sites received (1 star).

^b^ Association between age (1 star) or time on ART (1 star) and retention.

^c^ LFU defined (1 star), follow-up time minimum 12 months (1 star), reported transfers out (TO) (1 star)
