# Supplementary material for: Retention of HIV-Infected Children in the First 12 Months of Anti-Retroviral Therapy and Predictors of Attrition in Resource Limited Settings: A Systematic Review
Source: PLoS One. 2016 Jun 9;11(6):e0156506. doi: 10.1371/journal.pone.0156506 (PMC4900559; doi:10.1371/journal.pone.0156506)
Supplement: S1 Text — (DOCX) [file pone.0156506.s003.docx]

**S1 Text. Detailed Publication Search Strategy.**

**PubMed**

((antiretroviral therapy) OR agents, antiretroviral[MeSH Terms]) OR anti hiv agents[MeSH Terms]) OR highly active antiretroviral therapy[MeSH Terms]) AND (retention) OR retention[MeSH Terms]) OR loss to follow up) OR follow up) OR outcomes) OR attrition) OR adherence) OR mortality) OR efficacy) OR evaluation))) AND english[Language])) AND ("2002"[Date - Publication] : "3000"[Date - Publication]))) AND ((((pediatr*) OR paediatr*) OR child*) OR infant*))) NOT europe[MeSH Terms]) NOT australia[MeSH Terms]) NOT north america[MeSH Terms]

**Embase**

Search A: anti-retrovir* OR highly active anti-retrovir* OR HIV OR human immunodeficiency virus

Search B: retention OR los* to follow up OR los* to care OR outcome OR attrition

Search C: A AND B

Search D: child* OR pediatric OR paediatric OR infant

Search E: C and D

Limits applied to Search E: English language, human subject, years 2002-current
